# Prognosis of Biliary Atresia Associated With Cytomegalovirus: A Meta-Analysis

**DOI:** 10.3389/fped.2021.710450

**Published:** 2021-08-18

**Authors:** Yilin Zhao, Xiaodan Xu, Gengxin Liu, Fang Yang, Jianghua Zhan

**Affiliations:** ^1^Graduate College, Tianjin Medical University, Tianjin, China; ^2^Department of General Surgery, Tianjin Children's Hospital, Tianjin, China

**Keywords:** biliary atresia, cytomegalovirus, jaundice clearance, meta-analysis, prognosis

## Abstract

**Objective:** The etiology of biliary atresia is unclear, but viral infection has been implicated. The aim of the current meta-analysis was to investigate relationships between cytomegalovirus (CMV) and the prognosis of biliary atresia.

**Methods:** PubMed, Embase, the Cochrane Library, the China National Knowledge Infrastructure database, and Wanfang Data electronic databases were searched for eligible studies. Each relevant text was thoroughly reviewed and examined, including related papers in their reference lists.

**Results:** A total of nine studies including 784 patients were included in the analysis. Biliary atresia patients with CMV exhibited significantly lower jaundice clearance (odds ratio: 0.46, *p* < 0.0001; *I*^2^ = 15%, *p* = 0.31). There were no significant differences in the rates of cholangitis or native liver survival. CMV-pp65-positive biliary atresia patients had a significantly lower rate of jaundice clearance (odds ratio: 5.87, *p* = 0.003; *I*^2^ = 0%, *p* = 0.71) and a significantly higher rate of cholangitis (odds ratio: 0.21, *p* = 0.01; *I*^2^ = 0%, *p* = 0.43) than CMV antibody-positive biliary atresia patients.

**Conclusion:** Biliary atresia patients who were also infected with CMV had a poorer prognosis, particularly with respect to jaundice clearance. CMV status may influence the prognosis of biliary atresia. Clinicians should be able to routinely identify the subset of biliary atresia patients who are also CMV-positive, in order to improve native liver survival.

## Highlights

- The current systematic review and meta-analysis was performed to investigate relationships between cytomegalovirus (CMV) and the prognosis of biliary atresia (BA).
- BA patients with CMV had worse prognoses, particularly with respect to jaundice clearance.
- CMV-pp65-positive BA patients exhibited lower jaundice clearance and a higher incidence of cholangitis than CMV-Ig-positive BA patients.
- It is important for clinicians to detect CMV infection early *via* different methods in children with BA.

## Introduction

Biliary atresia (BA) is a destructive and obliterative cholangiopathy that occurs in infants. It involves both the extra-hepatic and the intra-hepatic bile ducts, and it is characterized by progressive inflammation and fibrosis of the biliary tree (1). The incidence of BA ranges from 1/5,000 to 1/20,000, and it is particularly high in Asian countries (2, 3). The main treatment for BA is currently

Kasai portoenterostomy (KPE), which re-establishes bile flow and prolongs the survival of the native liver (4, 5). The etiology of BA may be associated with immune dysregulation, inflammation, and virus infection, but it is still unclear (6, 7). Cytomegalovirus (CMV) affects BA. Evidence of CMV infection has been reported in 10–38% of infants with BA in series from England, Germany, Brazil, and Sweden, but up to 60% in China (4, 6, 8). In a series of studies, CMV-associated BA patients exhibited poor outcomes, but other studies have yielded inconsistent results. These inconsistences may be due to the relatively small sample sizes involved. The present study aimed to investigate outcomes in patients with CMV-associated BA after KPE.

## Materials and Methods

The present review and meta-analysis were conducted in accordance with the Assessing the Methodological Quality of Systematic Reviews checklist and the Preferred Reporting Items for Systematic Reviews and Meta-Analyses guidelines.

### Search Strategy

An extensive search was conducted for studies that investigated associations between CMV and BA. Data published up to December 2020 were sourced from the PubMed, Embase, Cochrane Library, China National Knowledge Infrastructure, and Wanfang Data electronic databases. All relevant reports were thoroughly reviewed, including those in reference lists. The search terms were (Biliary Atresia) OR (Atresia, Biliary) OR (Intrahepatic Biliary Atresia) OR (Atresia, Intrahepatic Biliary) OR (Biliary Atresia, Intrahepatic) OR (Biliary Atresia, Extrahepatic) OR (Atresia, Extrahepatic Biliary) OR (Extrahepatic Biliary Atresia) OR (Idiopathic Extrahepatic Biliary Atresia) OR (Familial Extrahepatic Biliary Atresia) OR (bile duct atresia) OR (biliary atresia) OR [“Biliary Atresia” [Mesh]] AND [“Cytomegalovirus Infections” [Mesh]] OR [“Cytomegalovirus” (Mesh)] OR (cytomegaloviruses) OR (Infections, Cytomegalovirus) OR (Cytomegalovirus Infection) OR (Infection, Cytomegalovirus) OR (Cytomegalic Inclusion Disease) OR (Cytomegalic Inclusion Diseases) OR (Disease, Cytomegalic Inclusion) OR (Diseases, Cytomegalic Inclusion) OR (Inclusion Disease, Cytomegalic) OR (Inclusion Diseases, Cytomegalic) OR (Cytomegalovirus Infections). The search was conducted without restrictions on language or year of publication.

### Study Selection and Data Extraction

The inclusion criteria were (1) studies that included CMV detection in serum or urine; (2) randomized clinical trials (RCTs) or prospective/retrospective studies comparing CMV^+^ BA and CMV^−^ BA; (3) studies that included at least one of the following outcomes: “clearance of jaundice,” “cholangitis,” “NLS in 2 years,” or “survival curve on NLS”; and (4) studies with scores ≥6. Case reports, meeting abstracts, review articles, letters, and opinions were excluded. Two investigators independently assessed the eligibility of the studies by reviewing titles, abstracts, and, when required, full texts. Differences were settled *via* discussion. The extracted data recorded included first author, year of publication, study type, number of patients, jaundice clearance and cholangitis data, rate of native liver survival (NLS) in 2 years, and NLS survival curves. CMV can be detected *via* several methods. In the present analysis, CMV^−^ BA was defined as all negative indicators in both blood samples and urine samples, and any deviation from that was defined as CMV^+^ BA. CMV-Ig-positive BA was defined as CMV antibody-positive but CMV-pp65-negative, and patients who were positive for CMV-pp65 were defined as CMV-pp65-positive.

### Statistical Analysis and Exploration of Heterogeneity

Stata SE15.0 was used for the analysis. Odds ratios (ORs) with 95% confidence intervals (CIs) were calculated based on the reported numbers of patients and events. Hazard ratios (HRs) with 95% CIs were calculated according to calculate lnHR and its variance by NLS survival curves (9). Mean differences (MDs) with 95% CIs were used for continuous outcomes. Differences of *p* < 0.05 were considered statistically significant. Heterogeneity was assessed *via* the Cochrane Q test and *I*^2^ values (10). *p* < 0.1 or *I*^2^ < 50% was considered to indicate low heterogeneity and then a fixed effects model was used; otherwise, a random effects model was used (10).

### Quality Assessment and Publication Bias

The quality of the studies included was evaluated *via* the Newcastle–Ottawa Scale (NOS) by two reviewers independently. Studies with NOS scores ≥6 were considered high quality and were included in analyses. Publication bias and jaundice clearance results were evaluated *via* Egger's test, but other parameters were not analyzed due to the small number of studies.

## Results

### Search Results and Study Characteristics

**Figure 1:**
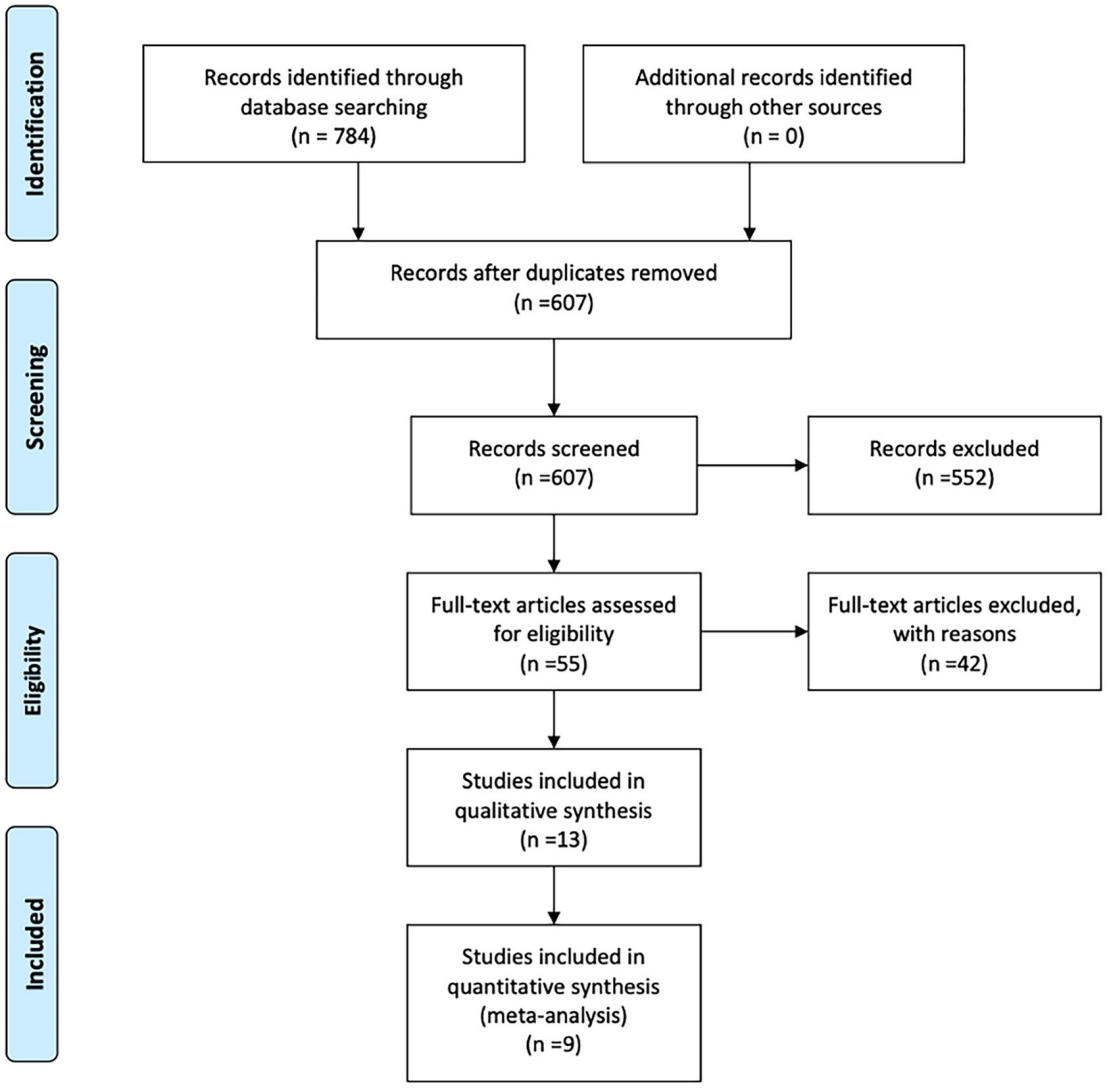
Flowchart of the study selection process.

A flowchart of the search results is shown in Figure 1. A total of 784 records were identified and evaluated by two investigators independently. After excluding 177 that were duplicates, a further 552 were excluded after screening the titles and abstracts. A further 42 studies were excluded due to no access to the full text or complete data. Nine studies published between 2007 and 2020 ultimately met the inclusion criteria and were included in the meta-analysis (6, 11–18). Detailed characteristics of the selected studies are summarized in Table 1. Eight of the studies included were retrospective and one was prospective, and collectively they included a total of 694 BA patients. Of these, 294 were classified as CMV^+^ BA patients. Characteristics of the reports included are shown in Tables 1, 2.

**Table 1:** Characteristics of the reports included in the meta-analysis.

**Reference**	**Year**	**Study type**	**Jaundice** **clearance**		**Incidence of cholangitis**		**2-year native liver survival**		**CMV** ^****+****^ **sample**		**CMV^**−**^ sample**	**NOS score**
			**exp**	**control**	**exp**	**control**	**exp**	**control**	**CMV-Ig^**+**^**	**CMV-pp65^**+**^**		
Zani et al. (6)	2015	Pro	3/20	57/109	–	–	–	–	20		109	7
Chun et al. (12)	2008	Re	13/22	4/5	6/22	1/5	–	–	11	11	5	6
Fischler et al. (11)	2008	Re	–	–	–	–	–	–	11		17	7
Song et al. (13)	2017	Re	22/35	80/123	–	–	20/35	69/123	35		123	6
Ji (16)	2020	Re	48/76	13/20	–	–	–	–	76		20	6
Lv et al. (15)	2019	Re	8/21	26/39	–	–	–	–	21		39	6
Qu et al. (17)	2013	Re	19/39	17/23	–	–	17/39	16/23	39		23	6
Luo et al. (18)	2007	Re	24/38	8/9	13/38	1/9	–	–	20	18	9	6
Dong et al. (14)	2016	Re	20/32	43/55	–	–	–	–	32		55	6
Total	–	–	–	–	–	–	–	–	294		400	–

*CMV, cytomegalovirus; exp, experimental; NOS, Newcastle–Ottawa Scale; Pro, prospective; Re, retrospective*.

**Table 2:** Data from the reports included in the meta-analysis.

**References**	**Detection of CMV-related BA**	**Jaundice clearance standards**	**Cholangitis standards**	**Treatment**
Zani et al. (6)	CMV IgM in serum	Bilirubin ≤ 20 μmol/L in 6 m	–	No antiviral drugs
Chun et al. (12)	One of CMV IgM, IgG, or pp65 in serum	TB < 34 μmol/L in 3 m	Within 6 m	–
Fischler et al. (11)	CMV IgM in serum or urine	–	–	No antiviral drugs
Song et al. (13)	Antibody in serum	Inexact definition in 3 m	–	–
Ji (16)	IgM in serum	Bilirubin < 20 μmol/L in 2 y	–	–
Lv et al. (15)	CMV DNA in serum > 500 copies/mL	TB < 34.2 μmol/L in 6 m	–	–
Qu et al. (17)	CMV antibody in serum	Bilirubin < 20 μmol/L in 6 m	–	–
Luo et al. (18)	One of IgM, IgG, or pp65 in serum	TB < 34 μmol/L and DB < 17 μmol/L in 3 m	Within 6 m	–
Dong et al. (14)	IgM	In 6 m	–	No antiviral drugs

*BA, biliary atresia; CMV, cytomegalovirus; DB, direct bilirubin; m, months; TB, total bilirubin; y, years*.

### Jaundice Clearance

**Figure 2:**
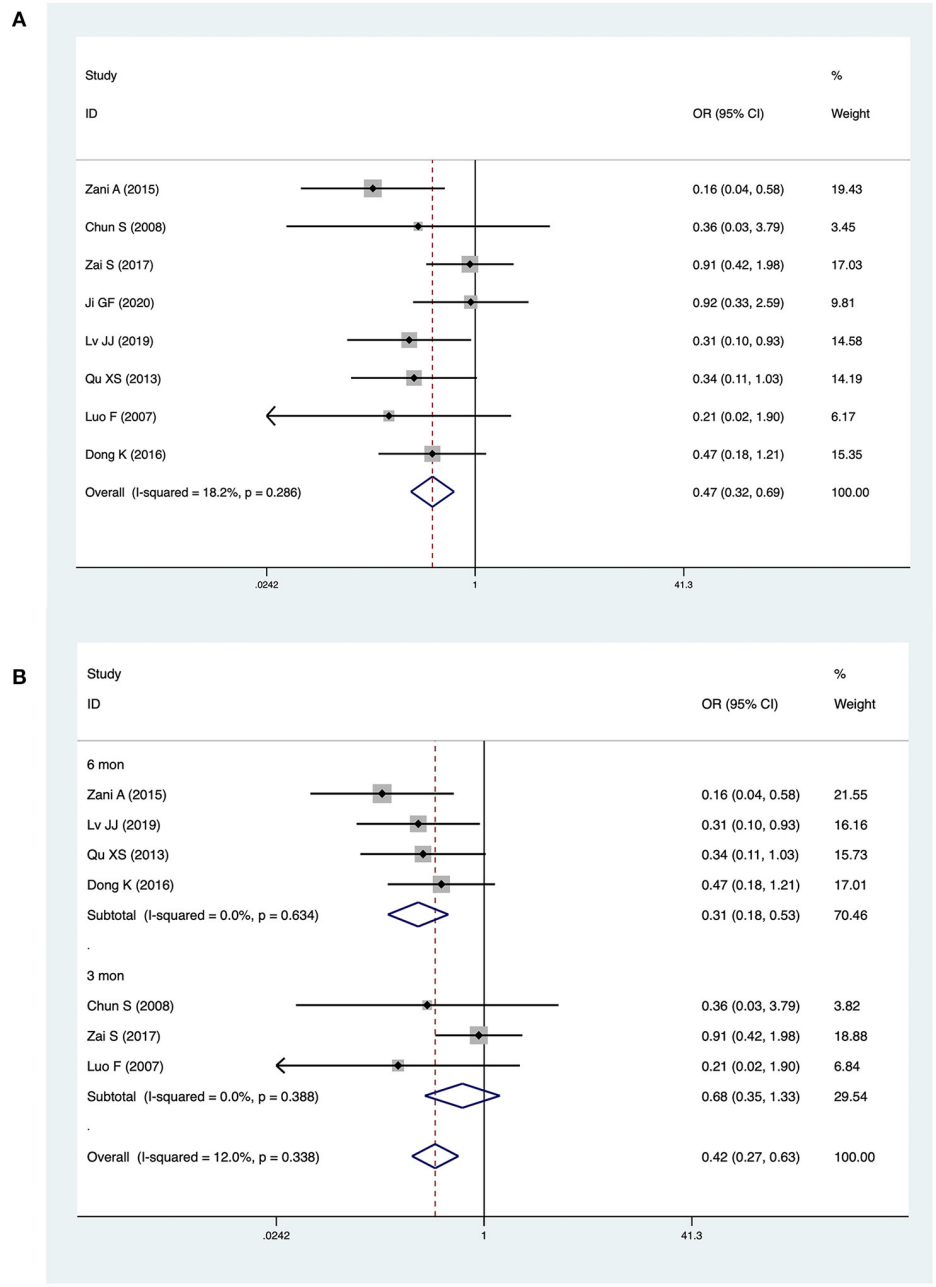
Forest plot of jaundice clearance in CMV^+^ BA vs. CMV^−^ BA **(A)**, in 6 months and 3 months **(B)**.

Eight studies with a total of 666 patients (283 CMV^+^ BA, 383 CMV^−^) described jaundice clearance. The pooled OR was 0.47 (95% CI: 0.32–0.69, *p* < 0.001) (Figure 2A). Heterogeneity among the studies was low (*I*^2^ = 18.2%, *p* = 0.286). Three studies reported jaundice clearance within 6 months. The pooled OR was 0.31 (95% CI: 0.18–0.53, *p* < 0.001), and heterogeneity was not significant (*I*^2^ = 0.0%, *p* = 0.634) (Figure 2B). Three studies reported jaundice clearance within 3 months (pooled OR: 0.68, 95% CI: 0.35–1.33, *p* = 0.256, *I*^2^ = 0.0%) (Figure 2B).

### Incidence of Cholangitis

**Figure 3:**
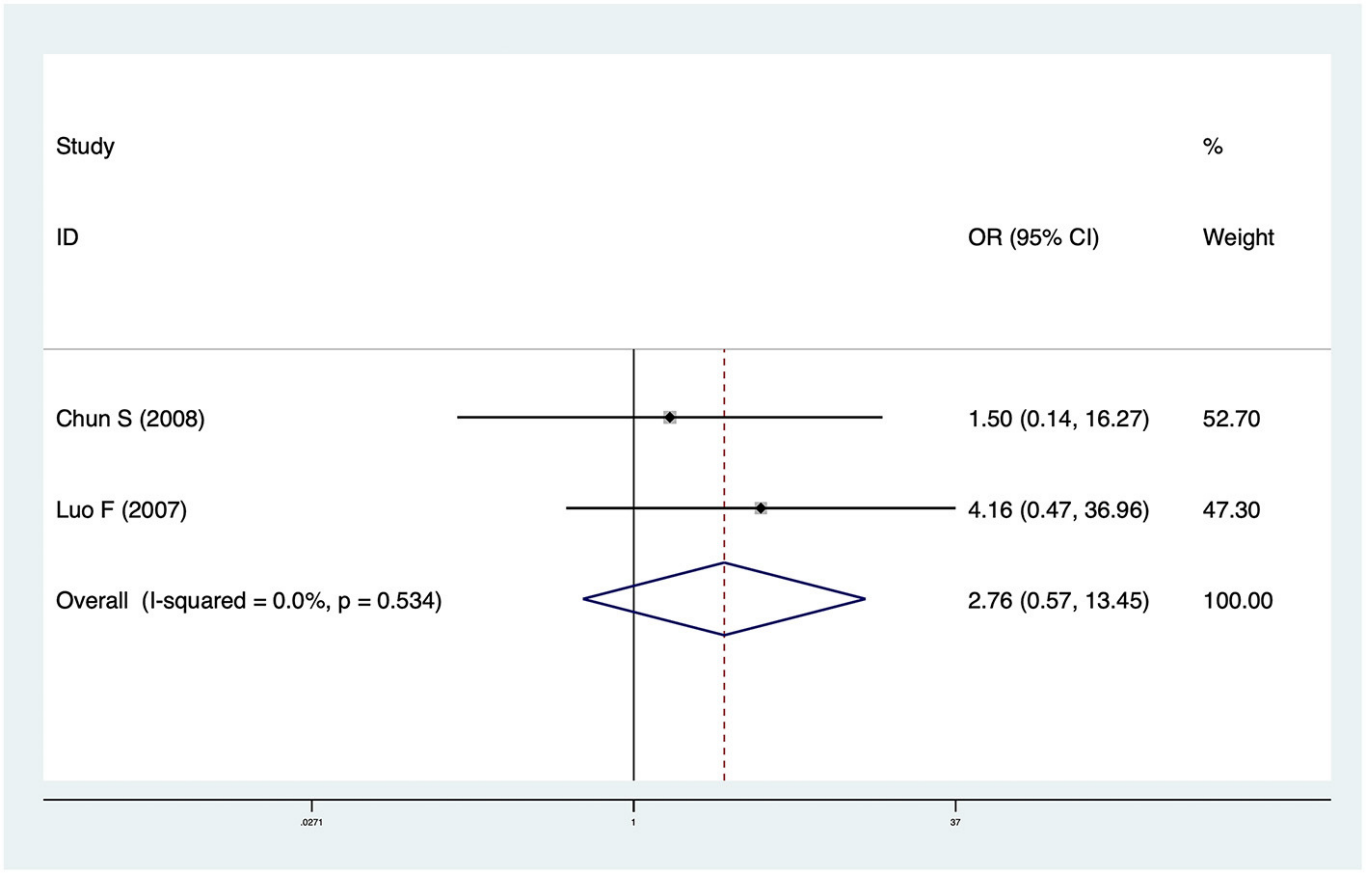
Forest plot of incidence of cholangitis in CMV ^+^ BA vs. CMV ^−^ BA.

Two studies including 74 patients reported cholangitis data. The pooled OR was 2.76 (95% CI: 0.57–13.45, *p* = 0.21) and heterogeneity was not significant (*I*^2^ = 0.0%, *p* = 0.534) (Figure 3). There was no significant difference in cholangitis incidence between CMV^+^ BA and CMV^−^ BA patients.

### NLS

**Figure 4:**
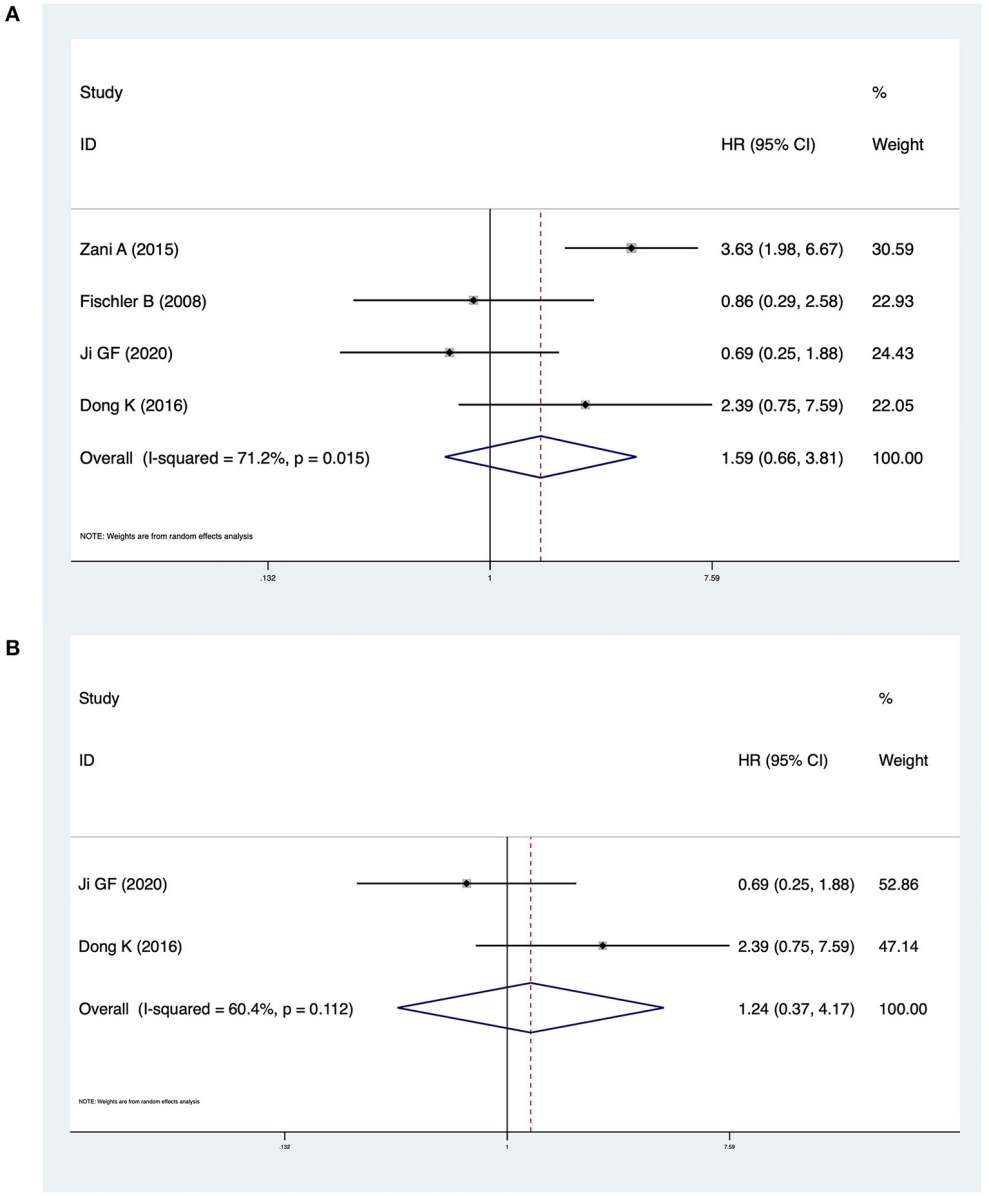
**(A)** Forest plot of the NLS in CMV ^+^ BA vs. CMV ^−^ BA. **(B)** Forest plot of the NLS in CMV ^+^ BA vs. CMV ^−^ BA in China.

Four studies including 340 patients reported NLS survival curves. The pooled HR was 1.59 (95% CI: 0.66–3.81, *p* = 0.301), and heterogeneity was significant (*I*^2^ = 71.2%, *p* = 0.015) (Figure 4A). In analyses restricted to studies conducted in China, there was no significant difference in NLS (HR = 1.24, 95% CI: 0.37–4.17, *p* = 0.729) and heterogeneity was high (*I*^2^ = 60.4%, *p* = 0.112) (Figure 4B).

### CMV-Ig and CMV-pp65 BA

**Figure 5:**
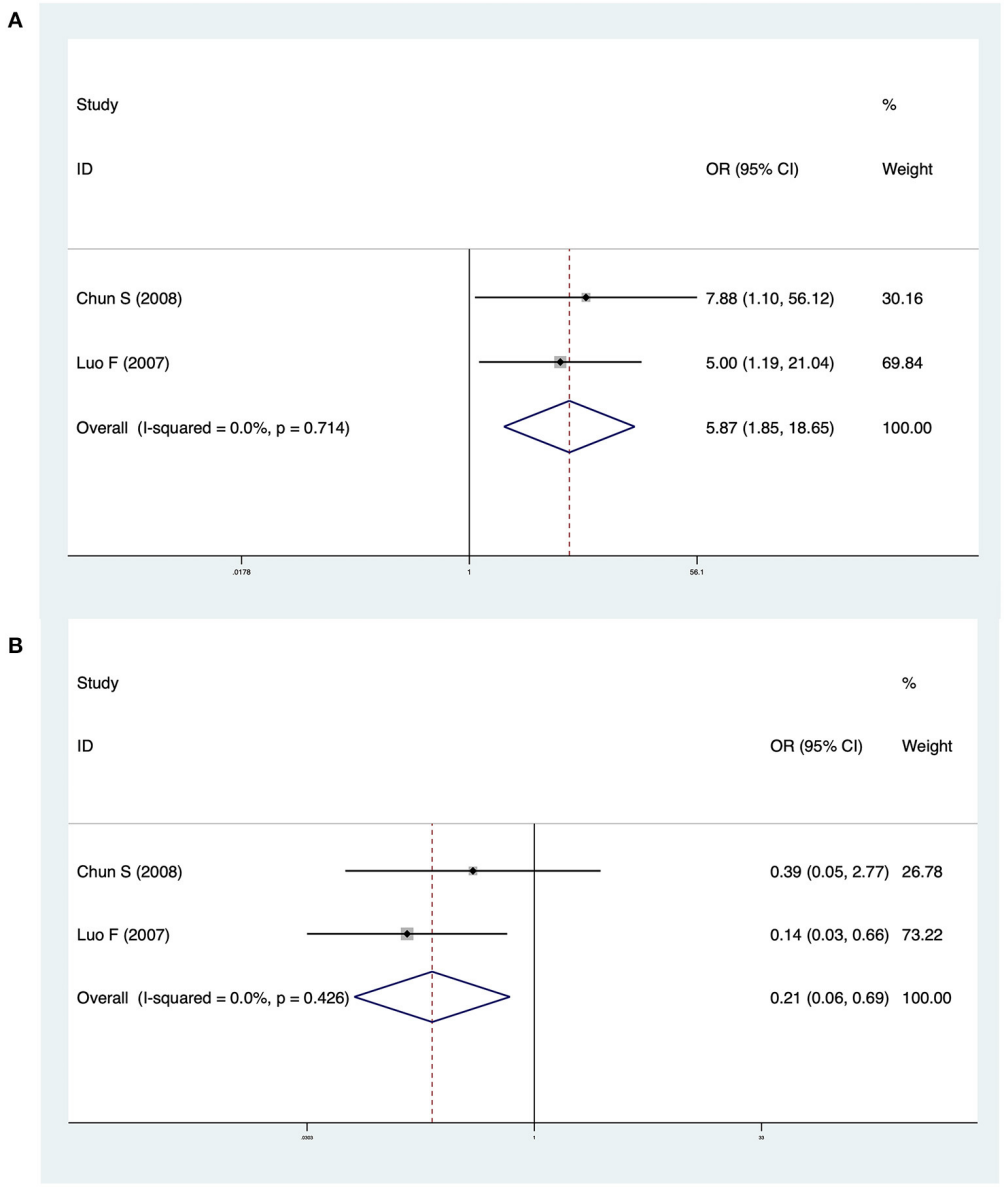
**(A)** Forest plot of jaundice clearance in CMV-Ig-positive BA vs. CMV-pp65-positive BA. **(B)** Forest plot of cholangitis in CMV-Ig-positive BA vs. CMV-pp65-positive BA

Two studies investigated differences between CMV-Ig-positive BA and CMV-pp65-positive BA. The pooled OR of jaundice clearance was 5.87 (95% CI: 1.85–18.65, *p* = 0.003), and heterogeneity was low (*I*^2^ = 0.0%, *p* = 0.714) (Figure 5A). The OR of cholangitis was 0.21 (95% CI: 0.06–0.69, *p* = 0.010), and heterogeneity was not significant (*I*^2^ = 0.0%, *p* = 0.426) (Figure 5B). In Zai et al. (13), CMV-pp65-positive BA patients exhibited significantly lower jaundice clearance and a significantly higher incidence of cholangitis than CMV-Ig-positive BA patients. Luo et al. (18) reported similar results.

### Others

**Figure 6:**
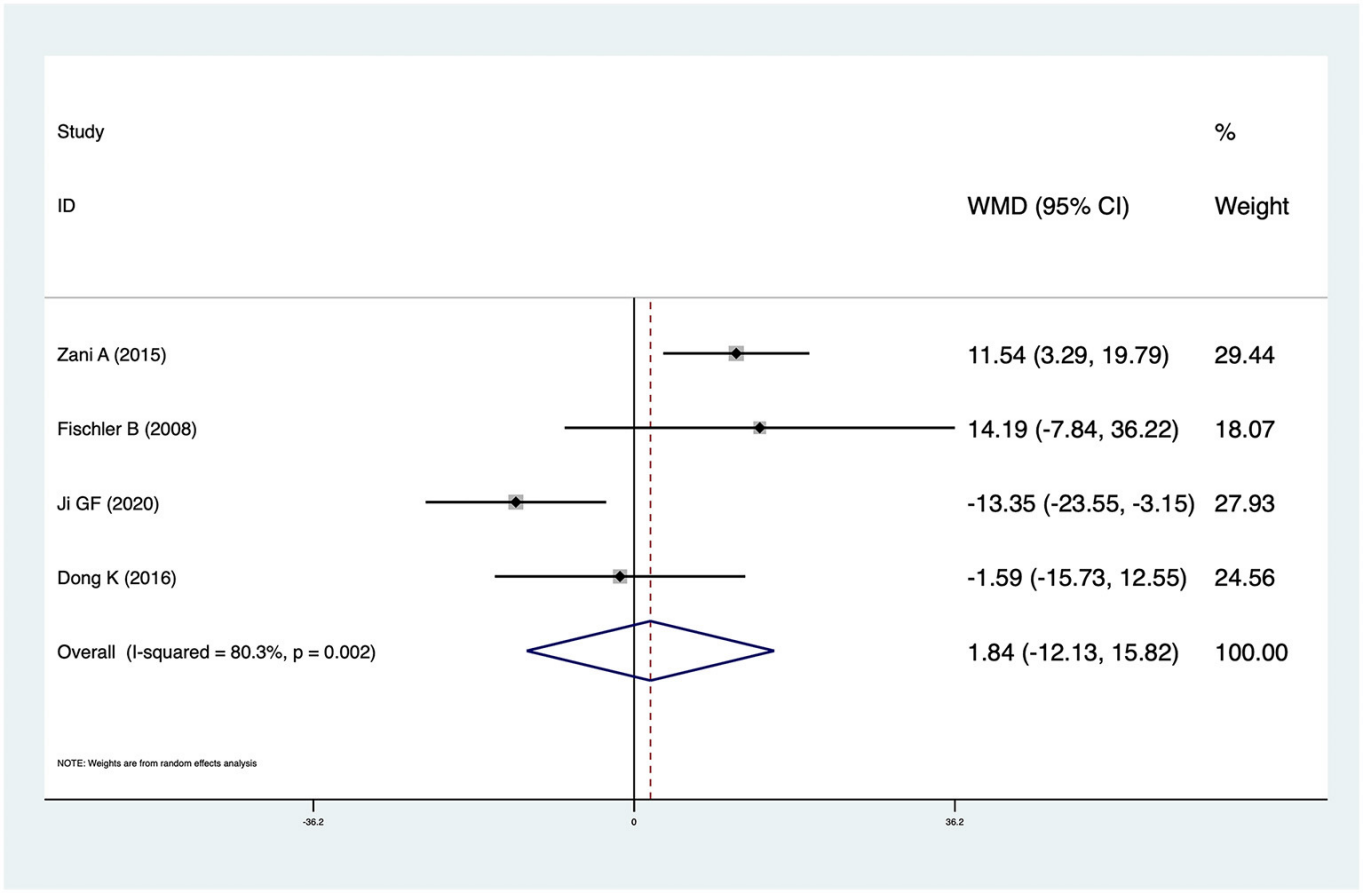
Forest plot of the KPE time of CMV^+^BA vs. CMV^−^BA.

Four studies reported KPE times in CMV^+^ BA and CMV^−^ BA patients. The pooled result was 1.84 (95% CI: −12.13 to 15.82, *p* = 0.796) and there was high heterogeneity (*I*^2^ = 80.3%, *p* = 0.002) (Figure 6). There was no significant difference between CMV^+^ BA patients and CMV^−^ BA patients. In Zani et al. (6), infants with CMV^+^ BA were significantly older at the time of KPE. In Dong et al. (14) and Fischler et al. (11), patients with CMV^+^ BA were older than those with CMV^−^ BA at the time of KPE, but not statistically significantly. In Ji et al. (16), patients with CMV^+^ BA were younger than those with CMV^−^ BA at the time of KPE, but not statistically significantly.

### Quality Assessment and Publication Bias

**Figure 7:**
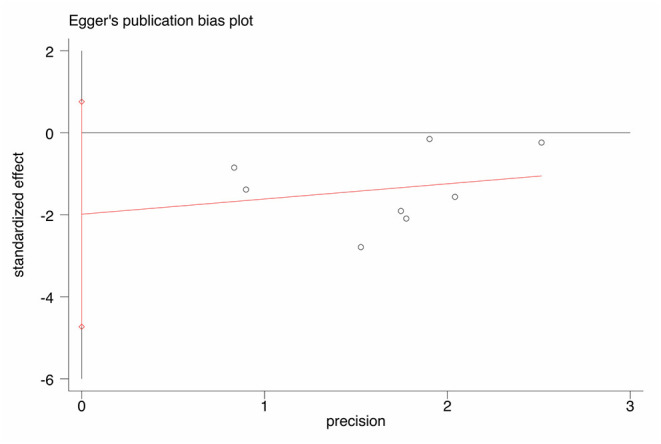
Egger's funnel plot for publication bias.

NOS scores are shown in Table 1. An Egger's funnel plot derived from the studies describing jaundice clearance in the current meta-analysis is shown in Figure 7. Egger's test indicated no significant publication bias (*t* = −1.77, *p* = 0.127).

## Discussion

CMV is a hepatophilic double-stranded DNA virus (6). Domiati-Saad et al. (19) reported that CMV was related to BA in a PCR-based study conducted in the 1990's, and Fischler et al. (20) detected CMV-IgM in serum in BA patients in the same era. In 2012, Davenport (3) identified CMV-IgM biliary atresia as a new subgroup. In a series of studies, CMV affecting the prognosis of BA was not limited to CMV-IgM positivity. Accordingly, in the present study, any positive indicator in blood or urine was used to define CMV^+^ BA, and a lack of any such indicators was used to define CMV^−^ BA. CMV^+^ BA is associated with a poor prognosis. In the present meta-analysis, patients with CMV^+^ BA had a significantly lower rate of jaundice clearance. Some studies reported that CMV^+^ BA patients presented symptoms later and were operated on later (6, 11, 16). CMV^+^ BA was associated with more severe fibrosis and inflammation during surgery than CMV^−^ BA (12, 14). There were indications that the virus triggered proinflammatory mechanisms, and this may lead to an autoimmune response guided by Th1 cells (21). At the time of KPE, liver fibrosis and inflammation were more severe in CMV^+^ BA patients than in CMV^−^ BA patients. There was a higher rate of cholangitis in CMV^+^ BA patients, but not statistically significantly. Cholangitis is one of the major complications of BA, and it is associated with a poor KPE prognosis. Repeated cholangitis can promote fibrosis, obstruct bile flow, and exacerbate jaundice and cirrhosis (22, 23). There was no significant difference in the rate of cholangitis in this study. The limited number of studies and the small sample sizes involved in the current investigation may have affected the results of the analysis. Different centers may also have different adjuvant therapies and different surgical operation levels, and this may also have affected the results of the analysis.

Various test measurements were used to detect the presence and status of CMV, including serum CMV-IgG, CMV-IgM, and CMV-DNA. CMV-pp65 can be detected in serum and urine. Positive CMV-IgM indicates recent infection that has been cured. CMV-IgG can be transferred through the mother's placenta and then it disappears gradually over the subsequent 2 years. CMV-pp65 antigenemia indicates duplication of CMV. CMV-DNA is an indicator of early active infection. In the present analysis, CMV-pp65-positive BA was associated with a poor outcome with respect to jaundice clearance and cholangitis. In Chun et al. (12) and Luo et al. (18), CMV-pp65-positive BA was associated with more rapid development of liver fibrosis than in other BA patients, including those who were CMV antibody-positive. CMV status may influence the outcome of BA. It is essential for clinicians to test for CMV status. Measurements derived from serum or urine do not represent the status of the liver. It remains a challenge to diagnose active CMV infection in the liver. Most pediatric surgeons agree that CMV is one of the etiologies of BA. CMV inclusion bodies have not been detected in the liver in any reported studies. Many problems remain to be solved with respect to CMV in BA.

Antiviral therapy has been proposed to improve the prognosis of CMV^+^ BA. In a study reported by Parolini et al. (24), antiviral drugs could negate the pathogenic effects of CMV, improve the rate of jaundice clearance, and reduce cholangitis. Notably however, there is not enough evidence to support the principle of adjuvant therapy, which currently depends on clinicians.

The present meta-analysis had several limitations. One was the lack of RCTs. Another was that the sample sizes of several of the studies included were relatively small. Some prognostic indicators were only included in a few of the studies analyzed, resulting in reduced statistical power and publication bias. No antiviral treatment was explicitly mentioned in any of the nine articles included. Lastly, there was a lack of comparisons of outcomes in CMV^+^ BA patients undergoing antiviral therapy vs. CMV^−^ BA patients.

In conclusion, the current meta-analysis indicates that CMV-IgM BA is a noteworthy subgroup of BA patients. BA patients with CMV exhibited poor prognosis, particularly with respect to jaundice clearance. CMV status may influence the prognosis of BA. Clinicians should be able to routinely identify CMV^+^ BA patients, to facilitate attempts to improve NLS.

## Data Availability Statement

The original contributions presented in the study are included in the article/supplementary material, further inquiries can be directed to the corresponding author/s.

## Author Contributions

YZ and FY contributed to the design of the research and perform statistics. YZ and XX contributed to design the statistical methods, search and choose studies, extracted the data, and pooled the results. YZ and GL interpreted the data, wrote and submitted the manuscript. JZ contributed to supervise the findings of this work, discussed the results, read and approved the final manuscript. All authors approved the final version to be published.

## Conflict of Interest

The authors declare that the research was conducted in the absence of any commercial or financial relationships that could be construed as a potential conflict of interest.

## Publisher's Note

All claims expressed in this article are solely those of the authors and do not necessarily represent those of their affiliated organizations, or those of the publisher, the editors and the reviewers. Any product that may be evaluated in this article, or claim that may be made by its manufacturer, is not guaranteed or endorsed by the publisher.
